# Supplementary material for: Content-rich biological network constructed by mining PubMed abstracts
Source: BMC Bioinformatics. 2004 Oct 8;5:147. doi: 10.1186/1471-2105-5-147 (PMC528731; doi:10.1186/1471-2105-5-147)
Supplement: Additional File 2 — The original results of the above study (non-essential files are deleted to keep the file size under the limit set by BMC bioinformatics). [file 1471-2105-5-147-S2.bz2 › chilibotAdditionalFile2/dip30/430ID7622448E10360/html/CCR1_CCL5.html]

 


 **CCR1** and **CCL5** 
  
Found 208 abstracts in PubMed, retrieved 30.  
 

 What does Google say? 
 PDF only 
| .edu only 

---

**Interactive relationship** (e.g. stimulation, inhibition, etc)

**Neutral relationship**- Oligomerization of RANTES  [ **CCL5** ]  is required for  **CCR1**  mediated arrest but not CCR5 mediated transmigration of leukocytes on inflamed endothelium.  Ref: 12763925 Blood< MedlineTA>Blood,
- Efficient leukocyte arrest in flow but not transmigration may thus require the presentation of RANTES  [ **CCL5** ]  oligomers to bridge surface bound RANTES  [ **CCL5** ]  and  **CCR1** .  Ref: 12763925 Blood< MedlineTA>Blood,
- Of seven  **CCR1**  and CCR4 agonists tested, only  **CCL5**  RANTES  [ **CCL5** ]  act as an agonist inducing chemotaxis.  Ref: 12270118 Biochem Biophys Res Commun, 2002
- Notably, the formation of higher order RANTES  [ **CCL5** ]  oligomers was crucial for  **CCR1**  mediated arrest but not for CCR5 mediated spreading transmigration in flow or transendothelial chemotaxis of leukocytes.  Ref: 12763925 Blood< MedlineTA>Blood, 2002
- Selective  **CCL5**  RANTES  [ **CCL5** ]  induced mast cell migration through interactions with chemokine receptors  **CCR1**  and CCR4.  Ref: 12270118 Biochem Biophys Res Commun, 2002
- Expression of L CCR  [ **CCR1** ]  in HEK 293 cells reveals functional responses to CCL2,  **CCL5** , CCL7, and CCL8.  Ref: 12885941 J Leukoc Biol, 2003
- One such receptor is  **CCR1**  which is a receptor for CC chemokines, such as  **CCL5**  RANTES  [ **CCL5** ]  and CCL3 MIP 1alpha.  Ref: 12769747 Curr Pharm Des, 2003
- These results demonstrate that both  **CCR1**  and CCR4 are functional receptors on human mast cells with capacity to mediate migration towards  **CCL5** .  Ref: 12270118 Biochem Biophys Res Commun, 2002
- However, the remaining expression of  **CCR1**  and CCR5 correlated reciprocally with an ongoing unresponsiveness to the  **CCR1**  and CCR5 agonists CCL3 macrophage inflammatory protein 1alpha MIP 1alpha, CCL4 MIP 1beta, and  **CCL5**  regulated on activation, normal T expressed and secreted RANTES  [ **CCL5** ] , all chemokines binding to these two receptors.  Ref: 12885942 J Leukoc Biol, 2003
- However, the specific functional relevance of RANTES  [ **CCL5** ]  oligomerization for distinct steps of leukocyte recruitment on inflamed endothelium mediated by the RANTES  [ **CCL5** ]  receptors  **CCR1**  and CCR5 remains undefined.  Ref: 12763925 Blood< MedlineTA>Blood, 2003
- The present findings suggest that MCP 1, RANTES  [ **CCL5** ] , and IP 10 may participate in the pathogenesis of mercuric chloride induced tubulointerstitial fibrosis with mononuclear cell infiltration, via CCR2,  **CCR1**  or CCR5, and CXCR3, respectively.  Ref: 12834626 Exp Mol Pathol, 2003

**Non-interactive relationship** (e.g. studied together, co-existance, homology, etc.)

- Only CpG ODN exacerbated HAF GN with an increase of glomerular macrophages, which was associated with massive albuminuria and increased renal MCP 1 CCL2, RANTES  [ **CCL5** ]   **CCL5** ,  **CCR1** , CCR2, and CCR5 mRNA expression.  Ref: 12538732 J Am Soc Nephrol, 2003
- Met RANTES  [ **CCL5** ]  treatment did not reduce CNS cellular infiltrates or up regulation of  **CCR1**  and CCR5 in affected CNS tissues.  Ref: 12098506 J Neuroimmunol, 2002
- To examine the function in EAE of murine types 1 and 5 CC chemokine receptors  **CCR1**  and CCR5, we used Met RANTES  [ **CCL5** ] , a peptide that blocks both receptors.  Ref: 12098506 J Neuroimmunol, 2002
- The expression of CXCR4 and  **CCR1**  and the migration to their ligands, SDF 1, and RANTES  [ **CCL5** ]  and MIP 1 alpha, respectively, were also demonstrated in primary MM cells.  Ref: 12529679 Leukemia, 2003
- Matching pairs of receptors ligands were found to be coordinately expressed, including TGFbetaRII with TGFbeta3, FGFRII and cysteine rich fibroblast growth factor receptor CRF 1 with FGF7 and FGF12,  **CCR1** , CCR3, CCR5 with RANTES  [ **CCL5** ]  and calcitronin receptor like gene CALCRL with adrenomedullin.  Ref: 12584245 J Cell Sci, 2003
- Although originally referred to as the MIP 1alpha RANTES  [ **CCL5** ]  receptor,  **CCR1**  is quite promiscuous and can be activated by numerous chemokines.  Ref: 12381680 Br J Pharmacol, 2002
- Astrocytes derived from mice with targeted mutations of either  **CCR1**  or CCR5 respond after RANTES  [ **CCL5** ]  stimulation, suggesting multiple chemokine receptors may separately mediate RANTES  [ **CCL5** ]  responsiveness in astrocytes.  Ref: 12112372 Glia, 2002
- Astrocytes expressed  **CCR1**  and CCR5 the redundant RANTES  [ **CCL5** ]  receptors.  Ref: 12112372 Glia, 2002
- In parallel,  **CCR1**  and CCR5 expression is down regulated and CXCL8, CCL2, CCL3 and  **CCL5**  production is up regulated.  Ref: 12548563 Eur J Immunol, 2003
- After 2 to 4 weeks, L. amazonensis infected mice had significantly delayed and depressed expression of inflammatory cytokines interleukin 12 IL 12, gamma interferon, IL 1alpha, IL 1beta, CC chemokines CC chemokine ligand 3 CCL3 macrophage inflammatory protein 1alpha MIP 1alpha, CCL4 MIP 1beta,  **CCL5**  RANTES  [ **CCL5** ] , MIP 2, and chemokine receptors  **CCR1** , CCR2, CCR5 in foot tissues and draining lymph nodes compared to the expression in L. major infected controls.  Ref: 12874303 Infect Immun, 2003
- Although only a minority of the cell lines responded by calcium mobilization after agonist stimulation, a migratory response to the  **CCR1**  ligands RANTES  [ **CCL5** ]  and MIP 1 alpha was obtained in 5 6 and 4 6, respectively, of the cell lines tested.  Ref: 12529679 Leukemia, 2003
- We used radioligand binding and 35S GTPgammaS exchange assays in membranes from a cell line transfected to express  **CCR1**  Ba F3 hCCR1 to characterize a panel of chemokines HCC 1, MIP 1alpha, MIP 1beta, MIP 1delta, MPIF 1, MCP 2, MCP 3, and RANTES  [ **CCL5** ]  as  **CCR1**  ligands.  Ref: 12381680 Br J Pharmacol, 2002
- Moreover, the forskolin mediated increases in intracellular cAMP concentrations were inhibited by the ligands for  **CCR1** , CCL3, CCL4, and  **CCL5** , suggesting that the expressed  **CCR1**  was functional.  Ref: 12651617 Am J Pathol, 2003
- To test the in vivo relevance of this, we used a murine melanoma system and knockout mice to investigate the function of the chemokine receptor CCR5 and its ligands, CCR  [ **CCR1** ]  ligand CCL 3 and  **CCL5** .  Ref: 12682253 J Immunol, 2003
- In J774 cells, CpG ODN induced the chemokines MCP 1 CCL2 and RANTES  [ **CCL5** ]   **CCL5**  and the chemokine receptors  **CCR1**  and CCR5.  Ref: 12538732 J Am Soc Nephrol, 2003
- Our studies showed that LD infection caused the upregulation of three beta chemokines macrophage inflammatory protein 1 alpha MIP 1alpha, MIP 1beta and RANTES  [ **CCL5** ]  regulated on activation normal T cell expressed and secreted, one alpha chemokine interleukin 8 IL 8 and the CC chemokine receptor 5 CCR5 but not  **CCR1** , as evident from reverse transcriptase polymerase chain reaction RT PCR analysis.  Ref: 12662300 Scand J Immunol, 2003
- Using 35S GTPgammaS exchange assays, intracellular calcium flux and or whole cell chemotaxis assays in HL 60 Rx cells, we validated that MIP 1alpha was the most potent  **CCR1**  ligand MIP 1alpha>MPIF 1>RANTES  [ **CCL5** ] >or=MIP 1beta although the ligands differed in their efficacy as agonists.  Ref: 12381680 Br J Pharmacol, 2002
- Furthermore, we also find that pretreatment with RANTES  [ **CCL5** ]   **CCL5** , the ligand for  **CCR1** , and CCR5 or SDF 1alpha CXCL12, the ligand for CXCR4, followed by opioid administration into the periaqueductal gray matter of the brain results in an increased rat tail flick response to a painful stimulus.  Ref: 12130663 Proc Natl Acad Sci U S A, 2002
